# X-linked recessive ichthyosis in 8 Tunisian patients: awareness of misdiagnosis due to the technical trap of the *STS* pseudogene

**DOI:** 10.1186/s12920-022-01319-4

**Published:** 2022-07-26

**Authors:** Hamza Chouk, Sarra Saad, Sarra Dimassi, Nadia Ghariani Fetoui, Ayda Bennour, Rima Gammoudi, Haifa Elmabrouk, Ali Saad, Mohamed Denguezli, Dorra H’mida

**Affiliations:** 1 Department of Cytogenetics, Molecular Genetic and Biology of Human Reproduction, Farhat Hached of Sousse, Sousse, Tunisia; 2 Higher Institute of Biotechnology of Monastir, Monastir, Tunisia; 3 Faculty of Medicine of Sousse, Sousse, Tunisia; 4 Department of Dermatology, Farhat Hached, Sousse, Tunisia

**Keywords:** STS gene, Contiguous genes, XLI, Ichthyosis, Genodermatosis

## Abstract

**Introduction:**

X-linked recessive ichthyosis (XLI) is a genodermatosis, caused by a deficiency of the steroid sulphatase enzyme encoded by the *STS* gene (OMIM # 300,747). Adopted XLI molecular diagnosis approaches differ from one laboratory to another depending on available technical facilities. Our work aims to figure out a sound diagnostic strategy for XLI.

**Patients and methods:**

We collected 8 patients with XLI, all males, from 3 unrelated Tunisian families from central Tunisia. Genetic diagnosis was conducted through a large panel of genetic techniques including: Sanger Sequencing, haplotype analysis of STR markers, MLPA analysis, FISH and array CGH.

**Results:**

Direct Sanger sequencing of the *STS* gene showed the same deletion of 13 base pairs within the exon 4 in all patients resulting in a premature stop codon. However, all patients’ mothers were not carriers of this variant and no common haplotype flanking *STS* gene was shared between affected patients. Sequence alignment with reference human genome revealed an unprocessed pseudogene of the *STS* gene located on the Y chromosome, on which the 13 bp deletion was actually located. *STS* MLPA analysis identified a deletion of the entire *STS* gene on X chromosome for all affected patients. This deletion was confirmed by FISH and delineated by array CGH.

**Conclusion:**

All our patients shared a deletion of the entire *STS* gene revealed by MLPA, confirmed by FISH and improved by array CGH. Geneticists must be aware of the presence of pseudogenes that can lead to XLI genetic misdiagnosis.

## Introduction

X-linked ichthyosis (XLI) (OMIM # 308,100) is the second most common type of ichthyosis after ichthyosis vulgaris. The first cutaneous symptoms appear during the first year of life. A slight appearance of a collodion baby or fine scaling may be seen at birth. However, the classic phenotype appears during early childhood [1]. This typical appearance of XLI is described generally as large, dark, polygonal or "dirty brown" scales covering the entire body and more prominent on the extensor legs [1].

XLI affects between 1/2000 and 1/6000 new born males according to the geographic locations, racial and ethnic background [2, 3]. It is caused by a deficiency of steroid sulfatase activity due to mutations in or deletions of the *STS* gene, located in the short arm of the X chromosome at Xp22.3. *STS* deficiency causes retention hyperkeratosis and impaired skin permeability [4].

To date, more than 71 different STS gene mutations have been reported in the XLI databases. Up to 90% of XLI are caused by a deletion of the entire *STS* gene, the remaining 10% result from partial deletions or point mutations [5, 6]. *STS* locus is too close to the pseudoautosomal region on X chromosome. It shares high sequence similarities with the pseudoautosomal locus on the Y chromosome. High deletion rate of the *STS* gene is due to the crossover between X and Y-chromosomes during male meiosis [7, 8].

Adopted XLI molecular diagnosis approaches differ between laboratories depending on the available technical facilities. Indeed, several molecular techniques such as fluorescent in situ hybridization (FISH), PCR, MLPA and array CGH enable the detection of *STS* gene deletions.

Herein, we describe the clinical features and genetic characteristics of 8 patients from 3 unrelated Tunisian families with XLI, and we highlight the similarities between the *STS* gene and its Y chromosome pseudogene sequences.

## Materials and methods

Informed consent was obtained from all the patients and parents of patients below 16 years of age belonging to 3 unrelated families. The study was approved by the ethical committee of Farhat Hached University Hospital of Sousse and was conducted following the Helsinki Declaration. A genetic study was performed on eight patients. The patient's genomic DNA was isolated from peripheral blood leukocytes by standard techniques, followed by molecular analysis of the extracted DNA by Sanger sequencing, haplotype analysis of STR markers, MLPA analysis, FISH and array CGH techniques.

### Primers design

The primers were designed using Primer 3 software (http://bioinfo.ut.ee/primer3-0.4.0/). All primer pairs were confirmed to be specific for the *STS* gene on chromosome X by database queries.

### Direct Sanger sequencing

Genomic DNA was extracted from peripheral blood leukocytes using FlexiGene DNA Kit by QIAGEN. The DNA was amplified by PCR of all exons and intron–exon boundaries of the *STS* gene and directly sequenced by Sanger method. The primers were designed to be specific for the *STS* gene using primer3 plus tools. The amplification conditions for the PCR consisted of one cycle of 94 °C for 5 min; denaturing at 94 °C for 30 s, annealing at 60 °C for 30 s, extension at 72 °C for 30 s, carried out for 33 cycles; and 72 °C for 10 min for the terminal extension. The sequencing products were separated by electrophoretic migration on the Applied Biosystems 3500 genetic analyzer. The sequencing results were subsequently analysed using SeqScape Version 2.7 software (AppliedBiosystems®).

### Haplotype analysis

Genotyping of 7 polymorphic microsatellites, including DXS1060, DXS8105, DXS996, DXS1223, DXS8051, DXS7103 and DXS7108 flanking the *STS* gene (http:// genome.ucsc.edu/), was performed by fluorescent PCR (primers and PCR conditions are available upon request).

### Multiplex ligation-dependent probe amplification (MLPA)

Multiplex Ligation-dependent Probe Amplification (MLPA) analysis, was performed using the commercial SALSA MLPA P160 C1 kit (MRC-Holland, Amsterdam, the Netherland) according to the manufacturer’s instructions. This probemix can also be used to detect STS deletions or duplications that extend into the neighbouring *ANOS1* (KAL1) and *NLGN4X* genes. Seventy nanograms of the genomic DNA were used for the MLPA reaction. The amplification products are separated and quantified on a genetic analyser (ABI 3500). The analysis and interpretation of the electrophoretic profiles were performed using GeneMarker® software. Thresholds to detect losses of genetic material were set, respectively, at 0.65 for heterozygous deletion in mothers and at 0.25 for hemizygous deletion in male patients.

### FISH procedure

The FISH procedure was performed using commercial probes, MD STS (Xp22) (Green signal)/KAL (Xp22) (Red signal)/SEX TC (Aqua signal) (Kreatech Diagnostics) on metaphase chromosomes from peripheral blood cells of one patient and his mother of each family. A drop of the probe applied to metaphases slides and co-denaturized for 7 min at 75 °C. After overnight hybridization at 37 °C, the slide was washed with a detergent solution to remove unspecific hybrids and unhybridized probes. Finally, chromosomes were counterstained with a 4′,6-Diamidino-2-Phenylindole (DAPI).

The analysis was carried out on an epifluorescence microscope: Axioscopt Zeiss®. A triple specific filter set appropriate for the fluorochromes available were used: FITC (fluorescein isothiocyanate, the signal emitted is green), rhodamine (the signal emitted is red), and chromosomes counterstained with DAPI (4′-6′ diamino-2-phenylindole), connected to a digital camera allowing the digitalisation of the images captured and their processing by an image analyser: Cytovision automated system, APPLIED IMAGIN®.

### Array CGH

In this study, two patients from two unrelated families underwent array CGH.

Agilent® 4X44K array was used according to the manufacturer's instructions (Feature Extraction 9.1, CGH Analytics 4.5, Santa Clara, California, United States). The average distance between two oligonucleotides is 44 kb providing an average resolution of 75,000 pb. The data extraction was performed using the Feature Extraction software and result interpretation with the CGH analytics 4.5 software. This software allows the analysis of the data extracted by Feature Extraction and gives the decimal log of the ratio between the fluorescence intensity of Cy5-labelled DNA and that of Cy3-labelled DNA. Therefore, a consensus threshold for recording an alteration was a copy number variation involving at least three consecutive oligonucleotides presenting an abnormal ratio greater than + 0.58 for duplications or lower than − 0.75 for deletions. An in silico analysis of the unbalanced region was made using UCSC Genome Browser (https://genome.ucsc.edu), the Database of Chromosome Imbalance and Phenotype in Humans using Ensemble Resources (https://www.deciphergenomics.org/).

## Results

### Clinical analysis

**Fig. 1:**
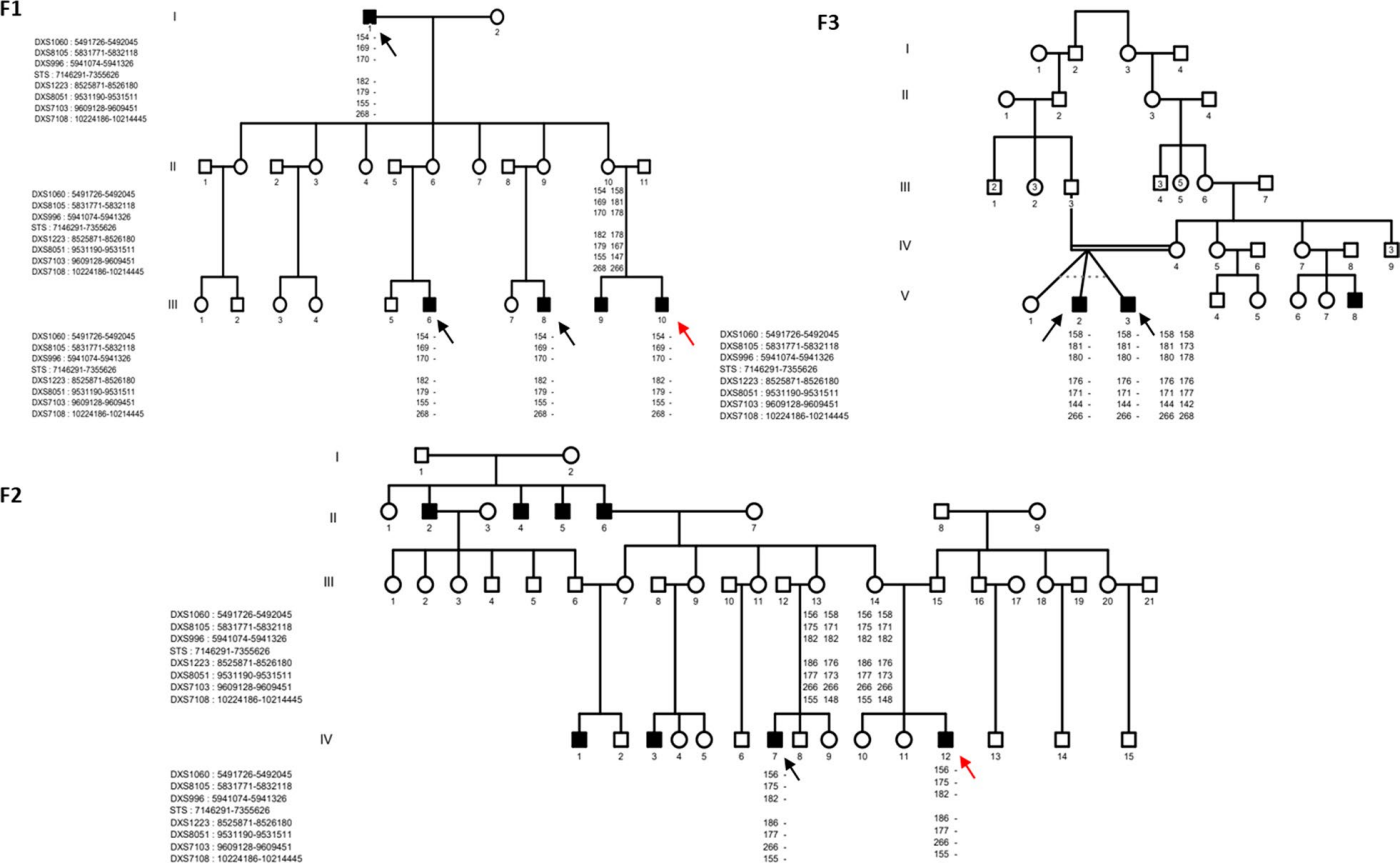
Pedigrees of the 3 Families (F1, F2, and F3): Haplotype study was performed in the 8 patients and in three of their mothers. Genotyping results for 7 microsatellites markers DXS1060, DXS8105, DXS996, DXS1223, DXS8051, DXS7103, and DXS7108 are shown. No common haplotype was shared by the three families. (The numbers in the male and female symbols in family 3 correspond to the number of siblings in the same generation. The red arrows correspond to patients who have undergone CGH array testing)

This study reports 8 patients belonging to three unrelated families from central Tunisia. All patients were male. The clinical manifestations appeared at birth in 4 patients of family F1 and during the first 3 months in patients of family F2 and F3 (Fig. 1).

Age at the first consultation ranged from 4 to 66 years. The clinical survey revealed the presence of cases of ichthyosis in the families of all our patients. All affected relatives belonged to the maternal branch of the index cases (Fig. 1).

**Fig. 2:**
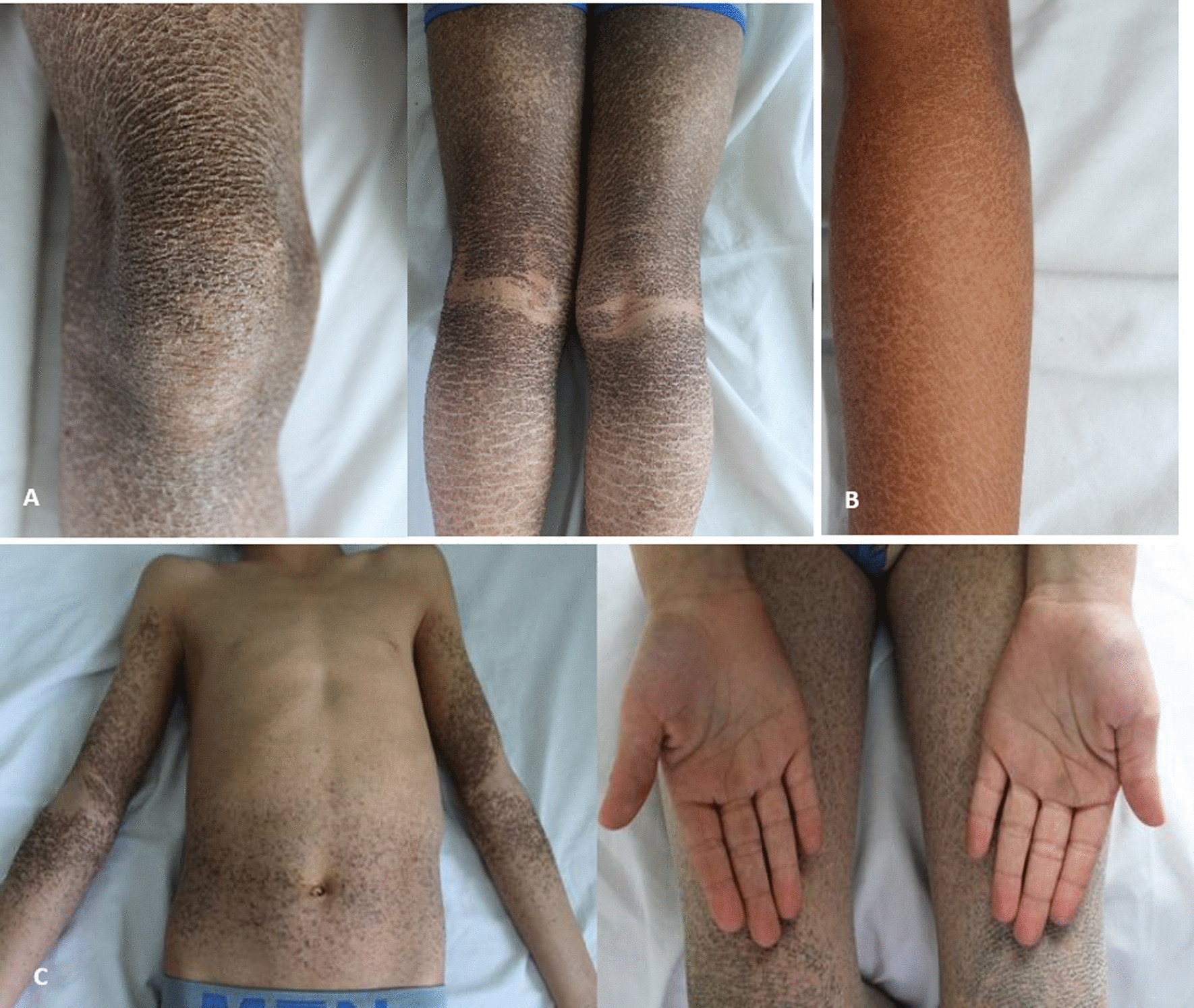
Dirty appearance of scales in patients from the three families’ characteristic of X-linked recessive ichthyosis. **A** Thick skin; **B** polygonal, adherent and large scales; **C** Prominent on the upper and lower limbs, thighs, and trunk

All 8 patients were born at term. The appearance of the skin at birth was normal in patients from families F2 and F3. The 4 patients from family F1 were born as collodion babies, however lacking ectropion and eclabion. Cutaneous examination showed thin and scaly skin in the 6 cases from families F1 and F3 and thick skin in the two patients from family F2 (Fig. 2a). The scales were polygonal and adherent in 7 patients and large in only one patient from family F1 (Fig. 2b). The colour of the scales was dark in 6 patients and brownish in 2 others of the family F1. The lesions were diffuse over the whole body in all cases. The lesions were prominent on the upper and lower limbs and the trunk in all patients (Fig. 2c). However, the folds were partially affected in all cases. The face and scalp were involved in 7 cases. Six patients had atopy and complained of pruritis. Erythroderma, palmoplantar keratoderma and palmar hyperlinearity were not observed in any of our 8 patients. One patient from family F3 underwent surgery for cryptorchidism. We noted an exacerbation of skin lesions during the winter season in all patients. Six patients were prescribed emollients and tropical urea with a favorable outcome.

### Molecular analysis

**Fig. 3:**
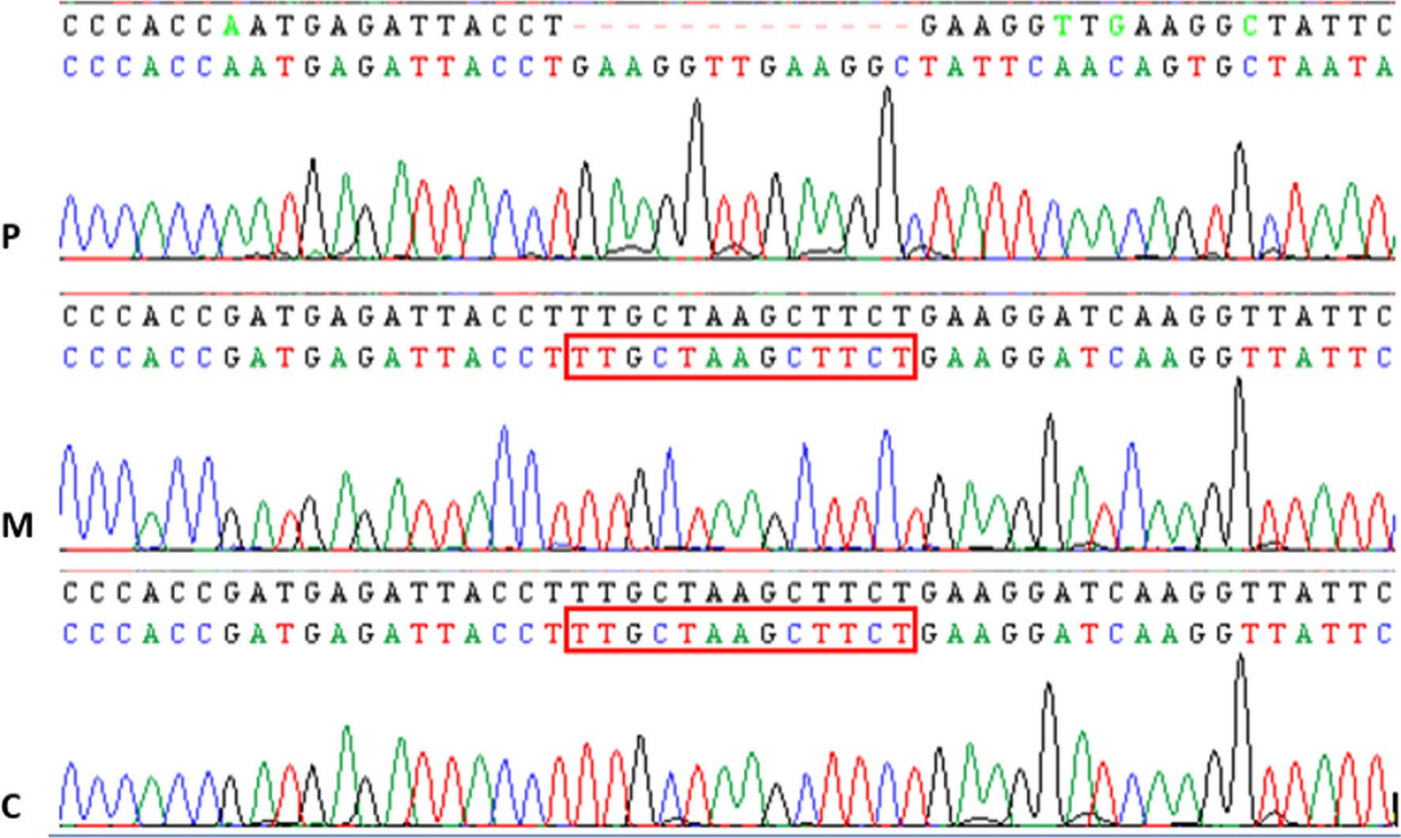
Sanger sequencing was performed in the 8 male patients and their respective mothers. The Sanger sequencing profile showed a deletion of 13 base pairs, in the 8 patients (P), that seems to be a new pathogen variant c.353_365del:(p.F118X) within the exon 4 of the STS gene resulting in a premature stop codon compared to their mothers (M) and to a healthy control (C) whose didn’t showed the deletion even at the heterozygous state

**Fig. 4:**
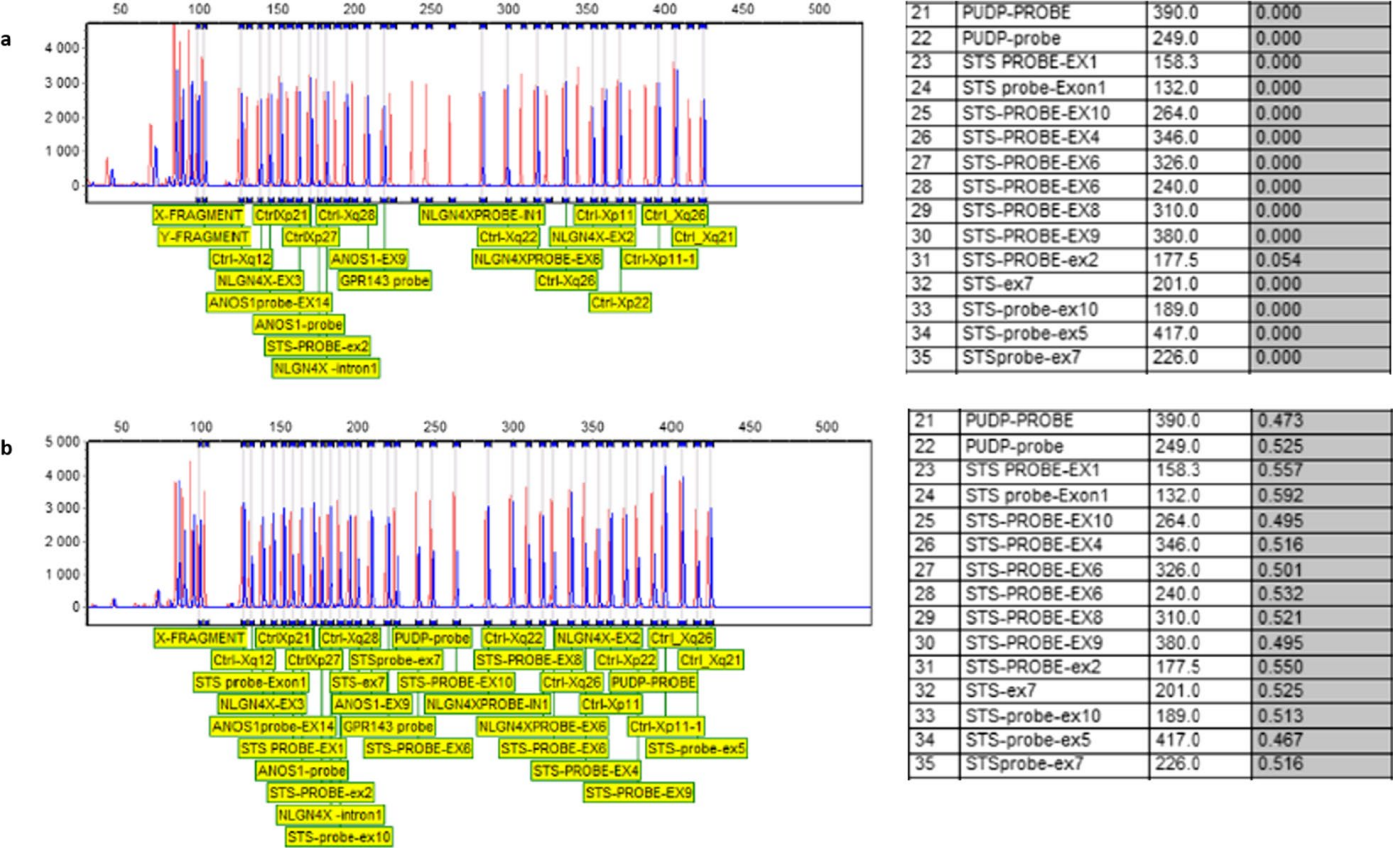
STS MLPA analysis; **a**: revealed a deletion of the entire STS gene for all patients of the three families, **b**: Mothers of the patients carrying the deletion in the heterozygous state. Thresholds to detect losses of genetic material were set, respectively, at 0.65 for heterozygous deletion in mothers and at 0.25 for hemizygous deletion in male patients

Direct Sanger sequencing of all our patients with diagnostic orientation of XLI identified the same 13 bp deletion c.353_365del:(p.F118X) within the exon 4, resulting in a premature stop codon of all patients. Nevertheless, this mutation does not segregate among mothers. Indeed, sequencing of exon 4 in all mothers did not reveal the presence of this mutation at heterozygous state (Fig. 3), and no shared haplotype flanking *STS* gene was noticed among affected patients (Figs. 1, 4).

**Fig. 5:**
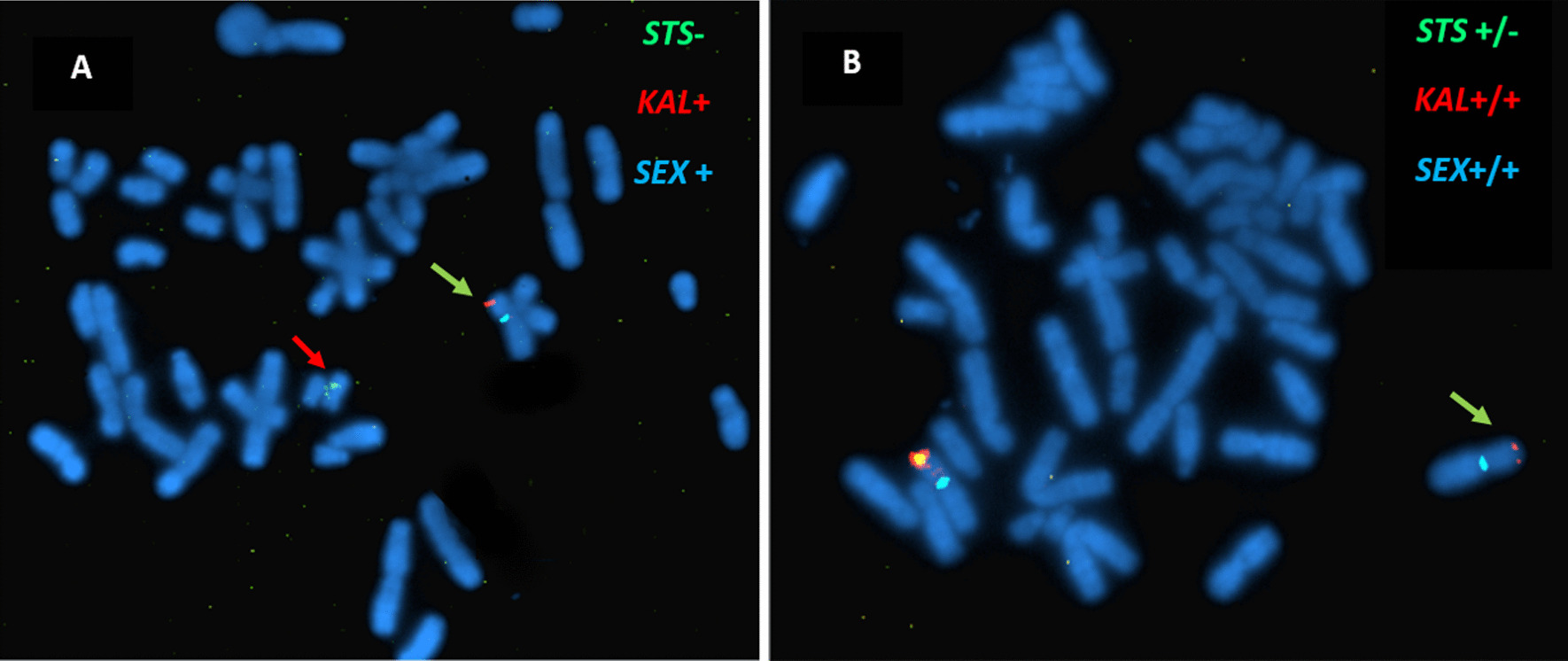
Metaphase fluorescence in-situ hybridization: (A, B) FISH analysis using STS probe (green signal), KAL1 probe (red signal) and centromere X probe (aqua signal); **A** FISH analysis of the proband III.10 from F1 family, showing STS gene deletion (green arrow) **B** FISH analysis of the mother's patient II.10., showing one copy deletion of the STS gene (green arrow). The proximity of the STS (green) and KAL (red) probes makes the presence of the two probes on an X chromosome in metaphase of the mother of patient III.10 of F1, is seen with yellow/orange coloration. A nonspecific hybridization of the STS probe (green) is also observed on the patient's Y chromosome (red arrow)

**Fig. 6:**
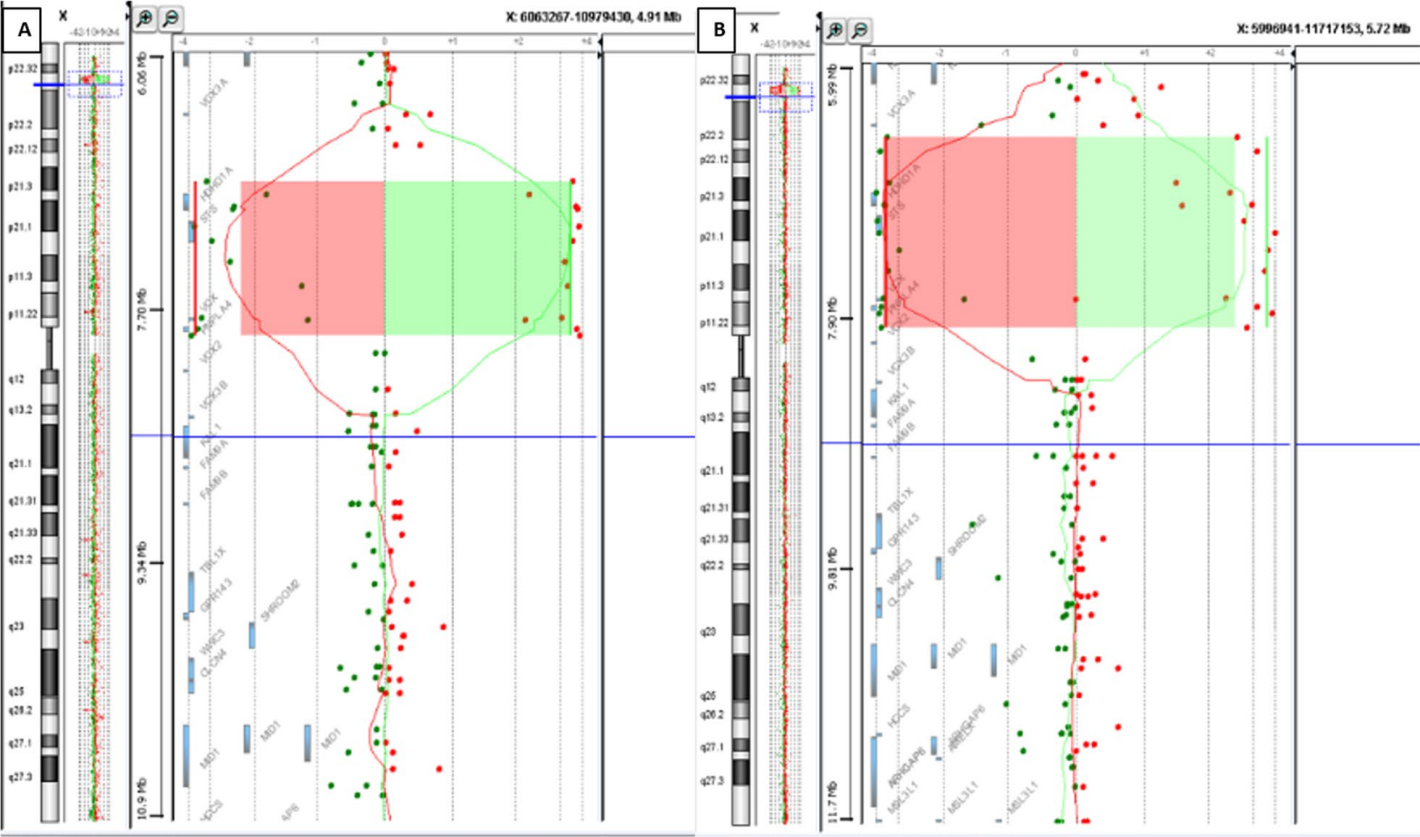
44 K Agilent array CGH results:** A**: Array CGH analysis of the patient III.10 from F1 family showing a deletion of 986.2 Kb at Xp22.31: arr [hg38] Xp22.31 (6,969,478_7,955,687) × 0; **B**: array CGH analysis of patient IV.12 from F2 family showing a deletion of 1.4 Mb at Xp22.31: arr [hg38] Xp22.31 (6,633,114–8,064,079) × 0

**Fig. 7:**
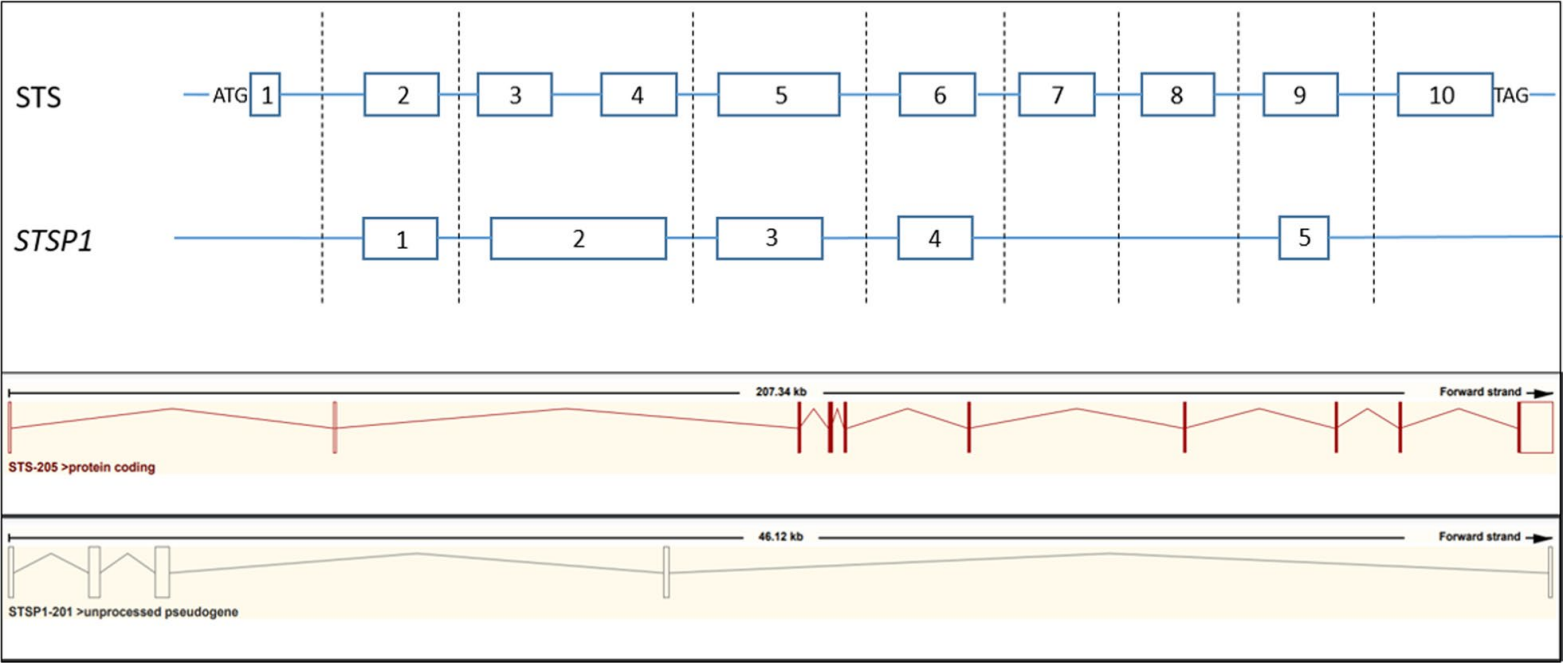
Structure of the STS gene versus the STSP1 pseudogene on the Y chromosome showing approximate similarity between the two genes. High sequence similarity between exon 2 of STSP1 and exons 3 and 4 of the STS gene, and no similarity of exons 1, 7, 8 and 10 of the STS gene with STSP1

FISH analysis revealed a deletion of *STS* gene on X chromosome in all 8 patients. Unspecific hybridization of S*TS* probe on Y chromosome was detected (Fig. 5A). FISH analysis of the mothers showing one copy deletion of the *STS* gene (Figs. 5B, 6). In fact, *STS* exon 4 patients’ sequence alignment with reference human genome revealed sequence homology with an *STS* unprocessed pseudogene on the Y chromosome (Fig. 7). MLPA analysis revealed a deletion of the entire *STS* gene for all patients (Fig. 4) and a heterozygous deletion in their mothers. *STS* entire deletion was best delineated by array CGH in patient III.10 from family F1 and patient IV.12 from family F2 (Fig. 6A and B respectively). Array CGH experiments identified a 986.2 Kb interstitial deletion in patient III10 from F1 family from oligonucleotide A_14_P139110 to oligonucleotide A_14_P131856 (chrX: 6,969,478–7,955,687, hg38) and a 1.4 Mb interstitial deletion in patient IV10 from F2 family from oligonucleotide A_14_P120930 to oligonucleotide A_14_P100326 (chrX: 6,633,114–8,064,079, hg 38). The two deletions share the same deleted OMIM genes: *STS and PUDP1*, *VCX*, *PNPLA4* (Fig. 6). The method used is the Dye Swap, comparing one patient against another. Each patient is taken once as control and once as a patient. This method has the advantage of the best attribution of the CNV, which appears in a mirror image.

## Discussion

X-linked recessive ichthyosis (XLI) (OMIM # 308,100) is the second most common type of ichthyosis after ichthyosis vulgaris. It affects approximately 1 in 6000 new born males from different ethnic groups and different geographic locations [2, 3]. In our report, we described 8 male patients from 3 unrelated Tunisian families. All patients showed a classical XLI phenotype, characterized by generalized, thick, dark and polygonal scales. Palms and soles are typically spared, which allows clinicians to distinguish XLI from ichthyosis vulgaris. Extracutaneous manifestations such as corneal opacity, mental retardation and gonadal alterations have been described in patients with XLI. One patient of our cohort has cryptorchidism.

*STS* gene is located on Xp22.3 close to the pseudoautosomal region. *STS* locus has the highest ratio of chromosomal deletion among all genetic disorders loci [5, 8]. Up to 90% of XLI patients display a large deletion of the entire *STS* gene. It is often deleted in XLI patients by unequal crossing-over. The presence of a high number of Low Copy Repetitive Elements (LCR), such as the hypervariable locus called (CRI232), flanking the *STS* and contiguous genes increases unequal crossing-over during female meiosis [5, 8]. Large deletions on Xp22.3 can spread to *STS* neighbouring genes and cause associated phenotypic abnormalities such as mental retardation, short stature and endocrine diseases [9, 10].

Patient III.10 from the F1 family and patient IV.12 from the F2 family showed two interstitial microdeletions in the Xp22.31 region of 986.2 Kb and 1.4 Mb, respectively. STS gene microdeletions are considered classic when the size ranges from 1.35 Mb to 1.6 Mb [9]. Regardless of different deletion breakpoints between the two patients, the latter share the same number of deleted OMIM genes that span the STS gene and PUDP1, VCX, PNPLA4 contiguous genes responsible for roughly similar clinical manifestations. In a previous Tunisian study, array CGH performed on one XLI patient showed an about 2 Mb deletion on Xp22.3 encompasses 6 OMIM genes including *VCX3A*, *HDHD1A*, *STS*, *VCX*, *PNPLA4* and *VCX2*. Deletion of *VCX3A* has been reported in association with an abnormal neurocognitive phenotype [10]. It was demonstrated by Van Esch et al. [11] that Deletion breakpoints are due to the presence of a high number of the CRI-S232 LCR sequence flanking the STS gene, each harbouring a member of the VCX gene containing several repeat units (RU2) located at the 3' end of the gene served as recombination sites. These findings may explain the different deletion breakpoints in our two patients.

MLPA and FISH analysis showed that the mothers of our patients are carriers of the same *STS* deletions. The mothers show no XLI clinical manifestations, confirming that the *STS* gene faces the "Lyonisation" theory in which it escapes X chromosome inactivation and maintains the enzymatic activity of *STS* in carriers’ mothers.

The high-sequence similarity; around 94% for exons; [12] between *STS* gene and the unprocessed pseudogene located on Yq11.2 (so-called *STSP1*) could mislead genetic analysis results (Fig. 7).

Herein, direct Sanger sequencing was performed for all our patients and revealed a 13 bp deletion within exon 4 of the X-chromosome *STS* gene. No common haplotype for the affected patients was detected around the X-chromosome *STS* gene, and no mother carried this mutation. In fact, instead of amplifying the *STS* gene sequences, the primers were amplified on the exon 2 of the *STSP1* pseudogene on the Y chromosome. The partial homology of the *STS* and *STSP1* genes (Fig. 7) explains the unstable amplification of exon 4 in our patients [13, 14]. The non-amplification of exons 1, 7, 8 and 10 in our patients is explained by their weak homology on Y chromosome.

## Conclusion

In this study, the clinical presentation and distribution of scales were identical in all 8 male patients. All patients have a large deletion that encompasses the entire *STS* gene with clear phenotype-genotype correlation. As the *STS* locus on Xp22.3 is considered to have the highest rate of chromosomal deletions of all genetic disorders, genetic diagnosis priority should be given to deletions that often cover the entire *STS* gene.

Geneticists must be aware of the presence of *STSP1* pseudogenes that can lead to misdiagnosis. Pseudogenes sequence similarities with the gene of interest must be taken into account when designing primers for sequencing and performing FISH analysis to avoid mistaken results.

According to our results, the appropriate diagnostic methods for XLI analysis are to start with large deletions using FISH, MLPA and CGH array techniques to eliminate 90% of possible large deletions, followed by Sanger sequencing to detect point mutations.

## Data Availability

The datasets generated or analyzed during the current study are included in this published article and its related files. Data supporting the manuscript can be requested from the corresponding author. The temporary deposition link for the data analysed during the current study: https://submit.ncbi.nlm.nih.gov/subs/variation_clinvar/SUB11350689/.
